# Tracing the *De Novo* Origin of Protein-Coding Genes in Yeast

**DOI:** 10.1128/mBio.01024-18

**Published:** 2018-07-31

**Authors:** Baojun Wu, Alicia Knudson

**Affiliations:** a Department of Biology, Clark University, Worcester, Massachusetts, USA; University of California, Berkeley

**Keywords:** DNA shuffling, *de novo* gene, GC content, parallel origins, purifying selection, yeast

## Abstract

*De novo* genes are very important for evolutionary innovation. However, how these genes originate and spread remains largely unknown. To better understand this, we rigorously searched for *de novo* genes in Saccharomyces cerevisiae S288C and examined their spread and fixation in the population. Here, we identified 84 *de novo* genes in S. cerevisiae S288C since the divergence with their sister groups. Transcriptome and ribosome profiling data revealed at least 8 (10%) and 28 (33%) *de novo* genes being expressed and translated only under specific conditions, respectively. DNA microarray data, based on 2-fold change, showed that 87% of the *de novo* genes are regulated during various biological processes, such as nutrient utilization and sporulation. Our comparative and evolutionary analyses further revealed that some factors, including single nucleotide polymorphism (SNP)/indel mutation, high GC content, and DNA shuffling, contribute to the birth of *de novo* genes, while domestication and natural selection drive the spread and fixation of these genes. Finally, we also provide evidence suggesting the possible parallel origin of a *de novo* gene between S. cerevisiae and Saccharomyces paradoxus. Together, our study provides several new insights into the origin and spread of *de novo* genes.

## IMPORTANCE

Emergence of *de novo* genes has occurred in many lineages during evolution, but the birth, spread, and function of these genes remain unresolved. Here we have searched for *de novo* genes from Saccharomyces cerevisiae S288C using rigorous methods, which reduced the effects of bad annotation and genomic gaps on the identification of *de novo* genes. Through this analysis, we have found 84 new genes originating *de novo* from previously noncoding regions, 87% of which are very likely involved in various biological processes. We noticed that 10% and 33% of *de novo* genes were only expressed and translated under specific conditions, therefore, verification of *de novo* genes through transcriptome and ribosome profiling, especially from limited expression data, may underestimate the number of bona fide new genes. We further show that SNP/indel mutation, high GC content, and DNA shuffling could be involved in the birth of *de novo* genes, while domestication and natural selection drive the spread and fixation of these genes. Finally, we provide evidence suggesting the possible parallel origin of a new gene.

## INTRODUCTION

New genes are the rich substrate of evolution that leads to various biological effects. The mechanisms giving rise to new genes can be placed into four categories (1): (i) gene duplication and rapid divergence, in which a new gene is derived from already existing genes in the same genome; (ii) horizontal gene transfer, in which a new gene is derived from already existing genes but from different genomes; (iii) an overprinting process, where mutations in a protein-coding gene allow the expression of a second protein-coding gene (2); and (iv) *de novo* origin, in which the noncoding region evolves to an open reading frame (ORF) through SNP and indel mutations (3, 4). Here, we refer to the fourth category as *de novo* gene, which is the focus of this study.

A *de novo* gene arising from a noncoding region was thought to be improbable (5, 6). In recent years, our knowledge of the distribution and function of *de novo* genes has been increasing since the first identification of *de novo* genes in *Drosophila* (7, 8). Until now, the *de novo* origins of species or lineage-specific protein-coding genes from noncoding DNA have been described in diverse lineages, including yeast, primates, and plants (3, 8–14). Compared to evolutionary conserved genes, *de novo* genes are overall shorter and have lower expression and tissue-restricted expression (14). The function of *de novo* genes is diverse. It has been shown that *de novo* genes can quickly become functionally important and essential for viability in *Drosophila* (15, 16). In primates, the few described *de novo* genes have been implicated in cancer and cancer outcomes (3, 17, 18).

Saccharomyces cerevisiae is one of the simplest eukaryotic organisms, with a relatively compact genome/gene content and a wealth of available phenotypic data associated with mutant and growth conditions (19, 20), which provide the chance to systematically study aspects of *de novo* genes. Previous studies of new genes in S. cerevisiae have helped shed light on their origin (11, 21, 22). To better understand their origin, spread, and fixation, *de novo* genes were sought in S. cerevisiae S288C using strict parameters, as in the analyses of Guerzoni and McLysaght (4). Through analyses of identified *de novo* genes between different species and among conspecific strains, we found multiple factors involved in the birth, spread, and fixation of these genes. In addition, we also suggest possible parallel origin of a new gene between different species.

## RESULTS AND DISCUSSION

### Detection of *de novo* genes in Saccharomyces cerevisiae S288C genomes.

Within the Saccharomycetaceae, S. cerevisiae S288C has the best annotated genome and massive phenotypic data under various mutant and growth conditions. For instance, the SPELL database from the Saccharomyces Genome Database (SGD, the most commonly used database in yeast) contains 537 data sets representing 11,889 total conditions (23). As in previous studies, we first compared the complete S. cerevisiae S288C proteome from the SGD database with that of 20 species from Saccharomycetaceae (see all strains in Materials and Methods). The S. cerevisiae S288C proteins that did not have significant sequence similarity (E value of 1 × 10^−4^) from the 20 species were regarded as initial *de novo* genes. It has been suggested that great variability in the estimates of new genes is partially due to sequencing gaps, annotation error, or gene loss. Recently, Moyers and Zhang (24) reported that using sequence similarity searching methods alone for identifying new genes commonly results in false positives. Although this conclusion is still debated (25), employing noncoding orthologous DNA in sister outgroups as a subsidiary parameter would be helpful to identify genuine *de novo* genes (4). Together with an expression cutoff FPKM (fragments per kilobase of transcript per million mapped reads) of ≥1.0, 84 *de novo* genes from S. cerevisiae S288C were identified (see Table S1 at http://baojunedisonwu.weebly.com/download.html), which have no protein hit in 20 species but have noncoding orthologous sequences in sister species Saccharomyces paradoxus CBS 432 and Saccharomyces mikatae IFO1815. All *de novo* genes overlap non-*de novo* genes, where there are 73 opposite strand overlaps and 11 same strand overlaps.

**FIG 1:**
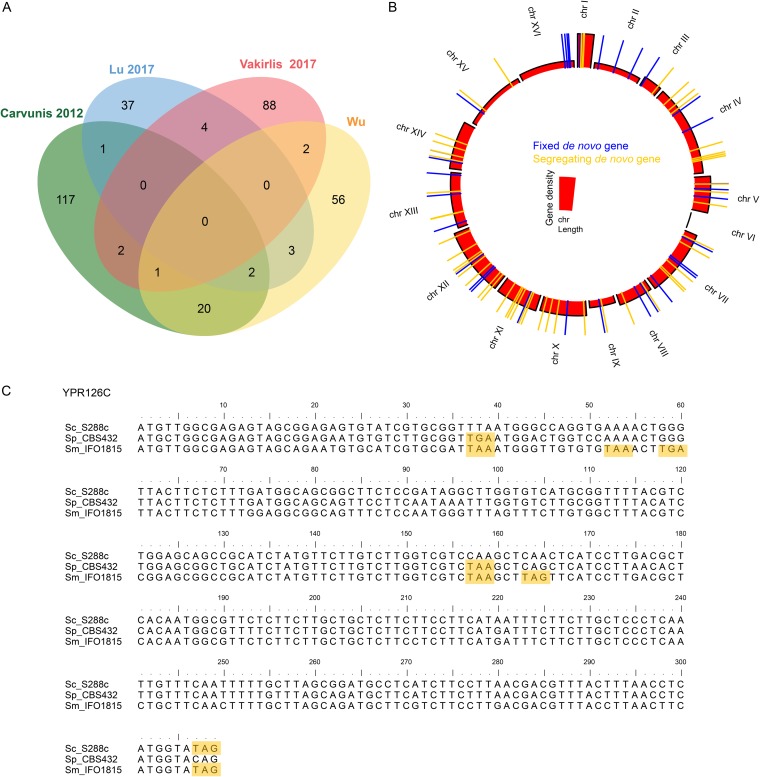
Eighty-four *de novo* genes detected in the S. cerevisiae S288C genome. (A) Overlaps of the *de novo* genes from this study and previous studies. Only SGD annotated genes were considered. (B) Distribution of 84 *de novo* genes along 16 chromosomes. (C) One example showing SNP mutations driving the birth of YPR126C. The yellow shading represents the position of the stop codon.

We compared our results with those of three other studies (11, 21, 22) (only considering the SGD annotated genes) and found that only 28 (33%) out of 84 genes were shared by the three other studies (Fig. 1A), in which 20 genes are found to overlap those of Carvunis et al. (11). Surprisingly, there is no common gene shared in all four studies (Fig. 1A). The variable results among studies can be partially attributed to exclusion of candidates overlapping ancient genes. For instance, ORFs overlapping ancient genes were often excluded from the Carvunis et al. study (11), but previous studies have indicated that new genes could arise while overlapping ancient genes (3, 4). In contrast, *de novo* genes overlapping ancient genes were considered in the Lu et al. study (22), but there are only five *de novo* genes shared between their work and this study (Fig. 1A). Therefore, besides the “overlapped” parameter, there are other parameters affecting the identification of *de novo* genes, such as E value for homologue searches and the required expression levels. For instance, in the Carvunis et al. study (2012), an E value of 10^−2^, more relaxed than the 10^−4^ used in this study, was used to search for homologues (BLASTP, TBLASTX, and TBLASTN). As a result, more annotated genes from S288C were found to have homologues in non-S. cerevisiae species than in this study. Moreover, whether homologues of S. cerevisiae S288C in non-S. cerevisiae species had intact open reading frames was not examined in the Carvunis et al. study (22). In other words, some homologues they identified in non-S. cerevisiae species are noncoding regions rather than protein-coding genes. Consequently, more true candidate genes than expected were filtered out. In the Lu et al. study (2017) (22), two copies of mRNA were used as the cutoff to further reduce the number of *de novo* genes. However, the study stated that there was a possibility that nontranscribed open reading frames might be expressed under other more specific conditions. Indeed, we found 10% of *de novo* genes were expressed only under specific conditions (see Table S1 at http://baojunedisonwu.weebly.com/download.html). Overall, among the four studies of yeast *de novo* genes, the numbers detected vary quite widely from study to study with very little overlap. A similar scenario was found in studies of primate *de novo* genes, where Guerzoni et al. (4) compared their results with those of Ruiz-Orera et al. (14) but found no overlap in the candidate lists.

### Segregating and fixed *de novo* genes in S. cerevisiae S288C.

The 84 *de novo* genes are spread across 15 of the 16 chromosomes (except chromosome VI [chr VI]), where chr I has the highest density (highest height) with 22 *de novo* genes per megabase (Fig. 1B). Based on a previous study (27), we inferred the boundary of core regions in each chromosome (see Table S2 at http://baojunedisonwu.weebly.com/download.html) and found all *de novo* genes were located within the core regions. We compared the orthologues of *de novo* genes against a 93-strain population and found 63% (52/84) of *de novo* genes carry alleles having both intact and disrupted ORFs. Therefore, 84 *de novo* genes could be divided into two categories: fixed *de novo* genes and segregating *de novo* genes. The fixed *de novo* genes are species specific, and the segregating *de novo* genes are strain specific. There are 13 chromosomes having both categories, while two chromosomes (chr II and chr XVI) only have fixed *de novo* genes (Fig. 1B).

Indel and SNP mutations have been reported to contribute to the birth of *de novo* genes in humans relative to other primates (3, 4). Compared with S. paradoxus CBS 432 and S. mikatae IFO1815, our analyses reveal that 2% (2/84), 30% (25/84), and 68% (57/84) of *de novo* genes are driven by indel mutation, SNP mutation, and a combination of indel and SNP mutations, respectively. For instance, along the full length of the S288C gene YPR126C, there is no gap, but there are SNPs resulting in two and five stop codons in the similar nucleotide sequences from S. paradoxus and S. mikatae, respectively (Fig. 1C). It is noteworthy that nucleotides at positions 1 to 39 produce a 12-amino-acid-long protein in S. paradoxus and S. mikatae, while positions 40 to 159 produce a 39-amino-acid-long protein in S. paradoxus, which suggests that new genes could evolve *de novo* through short ORFs in nongenic sequences (11). Among conspecific strains, 42% (22/52), 52% (27/52), and 6% (3/52) of segregating *de novo* genes are attributed to indel mutation, SNP mutation, and a combination of indel and SNP mutations, respectively. Moreover, these mutations in 74% (38/52) of segregating *de novo* genes occurred at the same positions, indicating that the disrupted ORFs might be under slight selection during spread within a population.

### *De novo* genes are possibly involved in biological process.

**FIG 2:**
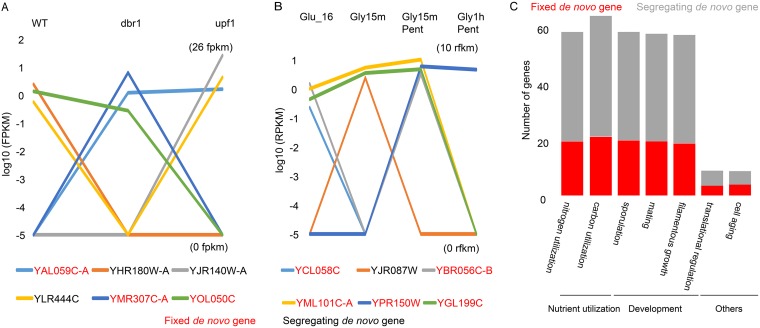
Expression, translation, and regulated evidence of *de novo* genes. (A) Dynamic expression of six *de novo* genes under different conditions. The FPKM values are in the range of 0 to ~26. All expression data of *de novo* genes are shown in Table S1. (B) Dynamic translation of six *de novo* genes under different conditions. The FPKM values are in the range of 0 to ~10. All footprinting data of *de novo* genes are shown in Table S4. (C) Various regulations of *de novo* genes associated with nutrient utilization, developmental stages, and cell aging. Red represents fixed *de novo* genes, and gray represents the segregating *de novo* genes. All regulation data of *de novo* genes are shown in Table S3.

The FPKM of *de novo* genes were extracted from previous transcriptome sequencing (RNA-seq) experiments (28) with the wild type and *dbr1*Δ and *upf1*Δ mutants, where the products of *dbr1* and *upf1* are two proteins involved in pre-mRNA splicing and nonsense-mediated mRNA decay (28, 29). Then, we used the FPKM value of ≥1.0 as the cutoff to filter out nonexpressed *de novo* genes (see Materials and Methods for more details). Among the expressed ones, we found eight *de novo* genes that were expressed only under specific conditions (six cases in Fig. 2A and for all cases in Table S1 at http://baojunedisonwu.weebly.com/), which suggests that these *de novo* genes could be regulated by *dbr1* and *upf1* and possibly involved in mRNA processing. It is important to note that both fixed and segregating *de novo* genes are among the eight *de novo* genes (six cases in Fig. 2A and for all cases in Table S1 at http://baojunedisonwu.weebly.com/); therefore, the potential function of *de novo* genes is not determined by their status (fixed or not) in the population. In addition, the condition-specific expression could result in underestimation of the number of *de novo* genes when expression level is used as a filter parameter, especially when only limited expression data are used. We further determined the translation of *de novo* gene using ribosome profiling data (30). This analysis identified 51% (43/84) of *de novo* genes having an RPKM (reads per kilobase of transcript per million mapped reads) value of ≥0.5, which was used as the cutoff of a translated gene (see more details in Materials and Methods). Among the 43 translated *de novo* genes, 28 genes were found to be translated at specific time points or conditions (six cases in Fig. 2B and for all cases in Table S3 at http://baojunedisonwu.weebly.com/). Similar to expression patterns, both fixed and segregating *de novo* genes could be translated under specific conditions.

We further took advantage of 537 expression microarray data from the SPELL database (23) to infer the potential function of expressed *de novo* genes. This compendium includes experiments sampled from a broad range of mutant and growth conditions. If a *de novo* gene had at least a 2-fold change relative to the control group on the microarray, it was regarded as a regulated gene. Among the 84 new genes, 73 (87%) were found to be associated with 52 functional categories defined by SPELL. For example, 61 genes (73%) are involved in the carbon utilization process, while only 6 genes (7%) are involved in cell aging (Fig. 2C). In addition, the proportion of segregating *de novo* genes in 46 categories is higher than that of fixed *de novo* genes (seven cases in Fig. 2C; see Table S4 at http://baojunedisonwu.weebly.com/download.html). Consistent with the observation that most *de novo* genes (87%) have potential functions, a recent study showed that expression of random artifact sequences with a coding region (50 amino acids) in bacteria could change cell growth rate, and the functional proportion of these random ORFs was up to 77% (31).

### GC content is important for birth of *de novo* genes.

**FIG 3:**
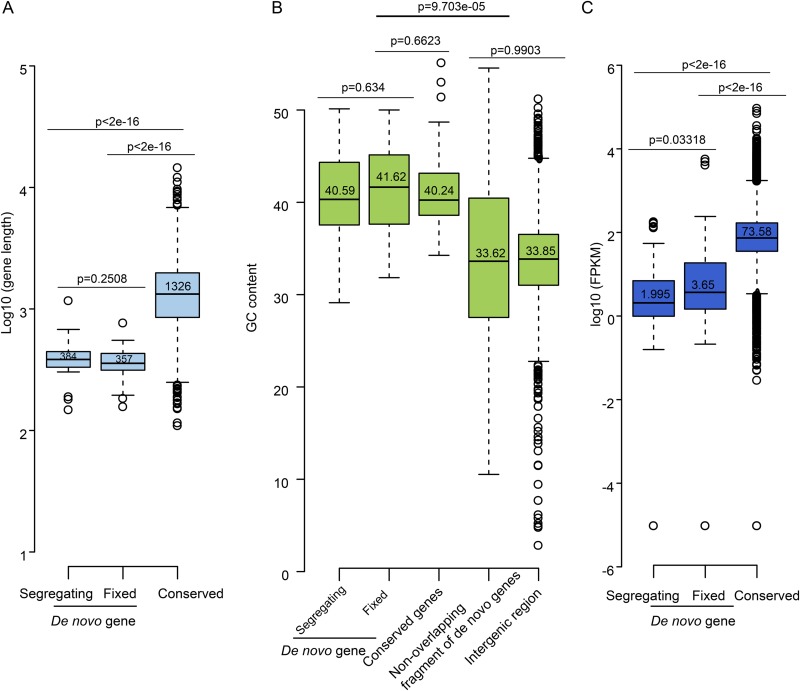
Comparison of *de novo* and conserved genes by (A) gene length, (B) GC content, and (C) gene expression level. For the intergenic regions, 4,539 fragments ranging from 200 to 1,000 nucleotides (nt) were used. The conserved genes were defined such that these genes are shared among S. cerevisiae S288C, S. paradoxus CBS432, and S. mikatae IFO1815.

In *Drosophila*, GC content, gene length, and expression level are positively correlated with sequence conservation (13), while new genes tend to be shorter, with low expression, and are GC poor. In agreement with studies in *Drosophila*, *de novo* genes of S288C are shorter (Fig. 3A) and are expressed at lower levels (Fig. 3C). However, different from *Drosophila*, where GC content of *de novo* genes is significant lower than that of conserved genes, the *de novo* genes in S288C have GC content no different from that of conserved genes (Fig. 3B). Given that all *de novo* genes overlap other preexisting genes, we also calculated the GC content of nonoverlapping regions, which shows obviously lower GC content than both conserved genes and full-length *de novo* genes but is similar to that in intergenic regions (Fig. 3B). This finding indicates that *de novo* genes formed as overlapping loci in high-GC regions associated with non-*de novo* genes. Moreover, these findings support the hypothesis that new genes are more likely to be generated from high-GC regions, while AT regions flanking GC regions act as the reservoirs for start and stop codons that define gene length. Generally, the GC content is important for the birth of a new gene because (i) GC-rich regions are more likely long ORFs by chance since stop codons are AT rich (32), (ii) GC-rich regions tend to be, on average, more transcriptionally active (33, 34), and (iii) higher GC content leads to higher intrinsic structural disorder (ISD) (35), which facilitates interprotein interaction and thus accelerates coadaptive evolution of new genes with preexisting genes. We also compared segregating and fixed *de novo* genes in terms of gene length, GC content, and expression level (Fig. 3A to C). However, only the difference in expression level was significant between them (*P* = 0.03), which is consistent with a previous observation from *Drosophila* (9), where the expression level of fixed *de novo* genes is higher than that of segregating *de novo* genes.

### DNA shuffling is a shortcut for sudden birth of a *de novo* gene.

**FIG 4:**
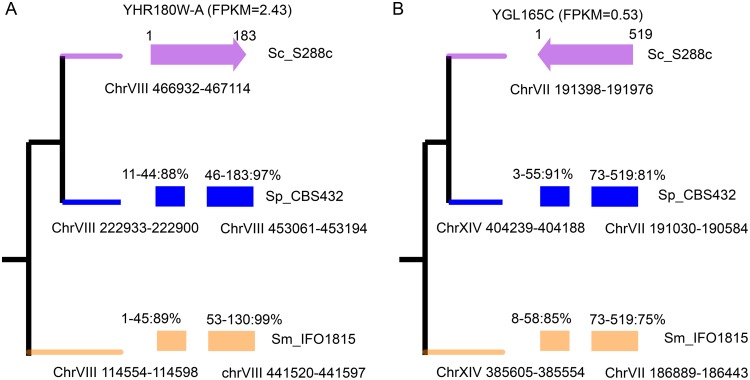
DNA shuffling shaping the birth of *de novo* genes (A) YHR180W-A and (B) YGL165C. The identity and corresponding region between *de novo* genes and noncoding DNA are labeled above the colored boxes. The genomic locations are labeled under the colored boxes. S. cerevisiae S288C is shown in pink, S. paradoxus CBS432 in blue, and S. mikatae IFO 1815 in cream.

Although birth of *de novo* genes can be driven by SNP and indel mutations in noncoding regions (3, 4), there are alternative evolution events driving their birth, such as DNA shuffling. There is one *de novo* gene, YHR180W-A, overlapping retrotransposon Ty3LTR. Given the mobility of transposons, it is reasonable to speculate that DNA shuffling may promote the birth of YHR180W-A over a short time. To test that possibility, we searched for the homologues from S. paradoxus CBS432 and S. mikatae IFO1815 using YHR180W-A as a query. As a result, we found YHR180W-A being shaped by the retrotransposon Ty3LTR and a tRNA-Thr, where the two features are separated by at least 200 kb in S. paradoxus CBS432 and S. mikatae IFO1815 (Fig. 4A). Previous studies indicated that 53% of primate new genes and 20% of human new genes match transposon elements (TEs) (14, 17). Therefore, the contribution of DNA shuffling mediated by transposons to the birth of new genes may be widespread. During the search for *de novo* genes, we also found that a *de novo* gene candidate was generated through DNA shuffling independent of a transposon, where two noncoding regions, shaping YGL165C, are located on different chromosomes from those of sister species (Fig. 4B). Although this candidate gene, YGL165C, does not meet the expression-level cutoff in this study (FPKM value of 0.53 versus 1.0), it provides insights into the birth of *de novo* genes mediated by DNA shuffling independent of transposons.

### Domestication and natural selection shape the spread and fixation of *de novo* genes.

**FIG 5:**
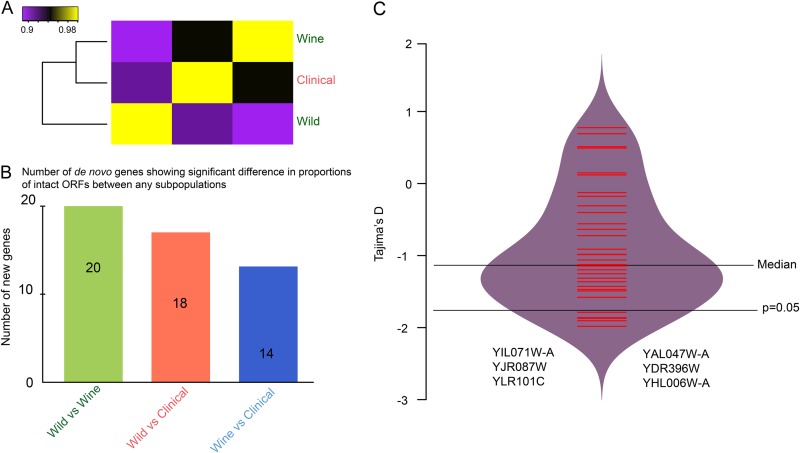
Domestication and natural selection of *de novo* genes. (A) Hierarchical clustering of subpopulations using the frequency of intact ORFs. (B) Number of *de novo* genes showing significant difference in proportions of intact ORFs between any two populations. (C) Inferred Tajima’s D values for the nonoverlapping regions from *de novo* genes. Only nonoverlapping regions longer than 50 bp were considered.

We divided the 93 strains into three subpopulations (wine-making strains, clinical strains, and wild strains) based on their environmental origins (see Table S5 at http://baojunedisonwu.weebly.com/download.html). Hierarchical clustering of subpopulations using the frequency of intact ORFs reveals that strains from the wine and clinical populations are closer to each other than the wild population (Fig. 5A). In addition, if the spread and fixation of *de novo* genes are free of selection, the frequency of intact ORFs for the same gene among three subpopulations should not be significantly different. However, we identified 29 genes showing a significant difference in the proportions between any two populations (Fisher’s exact test, *P* < 0.05) (Fig. 5B). Among the three pairwise comparisons, the combination of wine-making strains relative to wild strains has the most genes, while the pairwise comparison of wine-making versus clinical strains has the fewest genes. These observations suggest that domestication (environment) might play a role in the spread of *de novo* genes. We further investigated the role of natural selection through determining if *de novo* genes are associated with reduced nucleotide diversity (Tajima’s D). For each *de novo* gene, only the nonoverlapping regions with longer than 50 bp were considered, and then 36 genes (fragments) were collected for this analysis. Diversity in 6 of 36 genes (fragments) (17%) is significantly lower than expected (*P* < 0.05), and none of them is higher than expected (Fig. 5C). Moreover, all six fragments under purifying selection are from fixed alleles that do not have disrupted ORF alleles in the population, suggesting that natural selection plays a significant role in their fixation. Among the six genes (fragments), YIL071W-A is the only one among 16 new genes under purifying selection identified by Carvunis et al. (11).

### Possible parallel origin of *de novo* genes between species.

**FIG 6:**
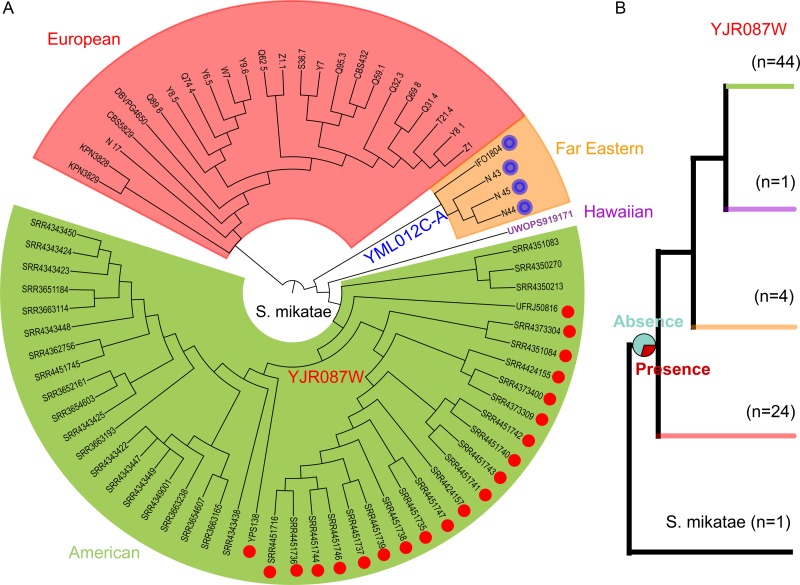
Parallel origin of a *de novo* gene. (A) Distribution of two S288C *de novo* genes in the S. paradoxus population. The maximum likelihood phylogeny was reconstructed using concatenated sequences of 1,000 aligned single-copy genes that are universally present in all S. paradoxus strains and the S. mikatae IFO 1815 strain (root species). Colors on the tree represent different subpopulations. (B) The ancestral states of YJR087W at the origin of S. paradoxus. The possibility is 70% absence and 30% presence, as shown in the pie.

We counted the new genes in 93 strains, but not in S288C and other yeast species, using the primary results from Strope et al. (2015) (36). Because there are no RNA-seq data for these strains, this analysis focused only on the birth of intact ORFs and resulted in the identification of 25 new genes, most of which (23/25) have a frequency of intact ORFs smaller than 10% (see Table S6 at http://baojunedisonwu.weebly.com/download.html). The alleles having both intact and disrupted ORFs in the S. cerevisiae population led to the question of possible misidentification of the *de novo* genes as a result of using only one strain from each outgroup species. To test that, we searched for orthologous loci from 73 S. paradoxus strains using 84 *de novo* genes (strains in Fig. 6) and found two targets from S. paradoxus carrying the alleles of both intact and disrupted ORFs, accounting for only 2% of the total *de novo* genes. Therefore, a single strain from each species as the outgroup would not greatly bias identification of *de novo* genes in this study. Among the two targets, 21 intact ORFs of YJR087W are present in a subpopulation of Ontario (red dots), while the intact ORFs of YML012C-A are found in the Far Eastern clade (blue circles) (Fig. 6A). Based on the distribution of intact ORFs, we inferred the ancestral states of the two genes and found that YJR087W had no (70% absence versus 30% presence) intact ORF at the origin of S. paradoxus (Fig. 6B; see Fig. S1 at http://baojunedisonwu.weebly.com/download.html). Given that the *de novo* gene YJR087W is present in a deep subpopulation of S. paradoxus and most of S. cerevisiae (Fig. 6A), this supports the possibility that a parallel origin of a *de novo* gene could occur between S. cerevisiae and S. paradoxus.

### Conclusion.

In this study, we identified 84 *de novo* genes (1% of total SGD genes) that originated recently in S. cerevisiae S288C. Analyses of these *de novo* genes reveal that SNP/indel mutations, high GC content, and DNA shuffling facilitate the birth of *de novo* genes, while domestication and natural selection play a role in the spread and fixation of these genes. In addition, our study also suggests a possible parallel origin of a *de novo* gene between S. cerevisiae and S. paradoxus.

## MATERIALS AND METHODS

### Identification of *de novo* genes in S. cerevisiae S288C.

We performed a BLASTP search of the S288C proteins downloaded from the Saccharomyces Genome Database (37) against the merged protein data set from S. paradoxus CSB432 (27) and 19 yeast species from YGOB (38) using an E value threshold of 1 × 10^−4^, which was used to identify new genes in other organisms (4). The 19 species include Saccharomyces mikatae, Saccharomyces kudriavzevii, Saccharomyces bayanus var. uvarum, *Candida glabrata*, Kazachstania africana, Kazachstania naganishii, Naumovozyma dairenensis, Naumovozyma castellii, Tetrapisispora blattae, Tetrapisispora phaffii, Vanderwaltozyma polyspora, Zygosaccharomyces rouxii, Torulaspora delbrueckii, Kluyveromyces lactis, Eremothecium gossypii, Eremothecium cymbalariae, Lachancea kluyveri, Lachancea thermotolerans, and Lachancea waltii. The genes not included in BLAST search results formed the basis for the list of initial candidate genes. We then excluded candidate genes where we could not detect the orthologous noncoding sequence in the outgroup genomes of S. paradoxus CBS432 and S. mikatae IFO1815. The *de novo* genes of S288C from three other studies were also extracted (11, 21, 22). In the Lu et al. study, the *de novo* genes were extracted from their Table S1. In the Vakirlis et al. study, the *de novo* genes were extracted from their Table S4. In the Carvunis et al. study, the *de novo* genes were extracted from their Table S3.

### Evidence of expression, translation, and regulation of *de novo* genes.

The FPKM of *de novo* genes were extracted from previous RNA-seq experiments (28), in which strand-specific libraries were constructed for three conditions: wild type and *dbr1*Δ and *upf1*Δ mutants. Previous study has proposed an FPKM value of 0.3 as the threshold separating intergenic and exon expression (39). In this study, an FPKM value of a *de novo* gene of ≥1.0 (3-fold as the threshold) under any condition was regarded as expression. Finally, 84 expressed *de novo* genes were identified and used for downstream analyses. We further determined the translation of *de novo* genes using the ribosome profiling data (30). In our study, the RPKM value of a *de novo* gene of ≥0.5 under any condition was thought to be translated. The RPKM value of 0.5 is reasonable because the ratio footprint RPKM value/RNA-seq FPKM = 0.5 is located within the normal range of translation efficiencies (30). Finally, we took advantage of 537 expression microarray data from the SPELL database (23) to infer the potential function of expressed *de novo* genes. If the expressed *de novo* gene had at least a 2-fold change relative to the control group on the microarray, it would be regarded as a regulated gene.

### Identification of alleles of S288C *de novo* genes from population genomes.

The 93 S. cerevisiae high-quality genomes were downloaded (with all accession numbers shown in Table S7 at http://baojunedisonwu.weebly.com/download.html); these were generated by Strope et al. (36). For the S. paradoxus genomes, we collected 31 strains from Liti et al. (ftp://ftp.sanger.ac.uk/pub/users/dmc/yeast/latest and ftp://ftp.sanger.ac.uk/pub/users/dmc/yeast/SGRP2/assembly/) (40) and Yue et al. (https://yjx1217.github.io/Yeast_PacBio_2016/data/) (27). In addition, we reassembled 42 strains from Xia et al. (41). The SRA accession numbers of the 42 strains can be found in Fig. 6A. *De novo* assembly was performed using SPAdes with four different k-mers (21, 33, 55, and 77) (42). In total, 73 S. paradoxus strains were collected. The 84 *de novo* genes from S. cerevisiae S288C were compared to the population data to identify their alleles. In particular, (i) a local BLASTN search was performed against population data using 84 *de novo* genes, (ii) hits were extracted from the population data, (iii) these extracted DNA sequences were aligned with *de novo* genes using MUSCLE (43), (iv) the alignments were manually checked based on reference *de novo* genes, (v) these refined alleles were translated into proteins using MEGA 7 (44), and (vi) stop codons were identified in the alignments of proteins.

### Analysis of selection on *de novo* genes.

The 93 strains were grouped into three subpopulations based on their environmental origins (see Table S5 at http://baojunedisonwu.weebly.com/download.html), and 12 strains with no clear categories were removed for this analysis. The different proportions of intact ORFs for *de novo* genes between any subpopulations were determined by Fisher’s exact test (*P* < 0.05). We also used the program DnaSP v5 (45) to calculate the population genetic parameters and to estimate deviation from neutral expectations for the nonoverlapping regions of the *de novo* genes.

### Reconstruction of ancestral state.

The 1,000 single-copy genes that are universally present in all examined 73 S. paradoxus strains and S. mikatae IFO1815 strains were used to construct phylogenetic relationships. Each gene was aligned individually using MUSCLE (43). The concatenated sequences of all gene alignments were used to reconstruct the phylogenetic relationship of S. paradoxus strains using the FastTree 2 program (46) under a general time-reversible (GTR) + Γ substitution model. The pattern of intact and disrupted ORFs was mapped on the phylogenetic tree of S. paradoxus population. Disrupted and intact ORFs at homologous sites were modeled as a two-state continuous-time Markov process, with states 0 and 1 on a phylogeny. The ancestral state for the *de novo* gene was then estimated using BayesTraits (47).

### Data availability.

The reassembled genomes of 42 strains from Ontario are available upon request.
